# *MyKid’sNutrition* mobile application trial: a randomized controlled trial to promote mothers’ nutritional knowledge and nutritional status of preschool children with undernutrition—a study protocol

**DOI:** 10.1186/s13063-023-07503-w

**Published:** 2023-08-19

**Authors:** Ali Hojati, Sogol Alesaeidi, Saeideh Izadi, Alireza Nikniaz, Mahdieh Abbasalizad Farhangi

**Affiliations:** 1 Tabriz Health Services Management Research Center, Tabriz University of Medical Sciences, Tabriz, Iran; 2 Department of Pediatric Medicine, Imam Hossein Hospital, Resident of Pediatric Medicine, Isfahan University of Medical Sciences, Isfahan, Iran; 3 Department of Surgery, School of Medicine, Laparoscopy Research Center, Shiraz University of Medical Sciences, Shiraz, Iran; 4 Department of Pediatrics, Faculty of Medicine, Tabriz University of Medical Sciences, Tabriz, Iran; 5 Department of Community Nutrition, Faculty of Nutrition, Tabriz University of Medical Sciences, Tabriz, Iran

**Keywords:** Preschool children, Undernutrition, Mobile application, Feeding practices, Maternal nutrition knowledge, Randomized controlled trial, mHealth

## Abstract

**Background:**

Childhood malnutrition is a crucial public health issue in developing countries. Mothers’ nutritional knowledge significantly affects children’s nutritional status. It also appears that mothers with low health literacy are unable to adequately meet their children’s nutritional needs because they do not clearly understand their children’s nutrition and malnutrition status.

**Methods:**

This randomized controlled trial aims to describe the development and study protocol of the *MyKid’sNutrition* application, a smartphone-based intervention for mothers of preschool (2–6 years old) children. The application contains several contents on children’s healthy eating, childhood underweight, children’s loss of appetite, and child growth assessment. As part of the *MyKid’sNutrition* trial, a total of 116 participants will be randomized 1:1 either to (a) treatment as usual and *MyKid’sNutrition* or (b) treatment as usual alone. The results of this trial will be based on changes in growth indicators and mothers’ nutritional knowledge, attitude, and practice within the groups and the differences between them.

**Discussion:**

Due to their widespread availability throughout society, smartphones can be used to deliver educational content on a large scale at a low cost. In addition, they can provide novel ways for patients to receive support. Hence, it is essential to conduct research studies on these types of interventions. *MyKid’sNutrition* application offers dietary solutions for such nutritional problems as underweight, loss of appetite, and malnutrition in children. Meanwhile, it provides detailed instructions on how to interact with the child.

**Trial registration:**

IRCT.ir IRCT20140907019082N11. Registered on February 19, 2022.

**Supplementary Information:**

The online version contains supplementary material available at 10.1186/s13063-023-07503-w.

## Introduction

### Background and rationale {6a}

In developing countries, childhood malnutrition continues to be a significant public health and development issue, and inequities in malnutrition persist [1]. Studies demonstrated that undernutrition during childhood has considerable economic consequences regarding children’s health and development, including restricted growth, diminished intelligence, weak cognitive development, and reduced productivity when they become adults [2, 3]. Numerous diseases are associated with malnutrition on a global scale, and undernutrition is responsible for about half of the annual deaths of children under 5 worldwide. In 2020, the World Health Organization (WHO) estimated that 149 million children under 5 were stunted, and 45 million were wasted [4].

Three commonly known indicators of a child’s nutritional state are stunting (low height for age), wasting (low weight for height), and being underweight (low weight for age). While underweight is a compound index encompassing acute and chronic malnutrition, stunting and wasting indicate chronic and acute malnutrition, respectively. However, different types of malnutrition can develop concomitantly in children [5]. Primary indicators of undernutrition-related malnutrition in cases where only one data point is available for diagnosis are as follows: weight for height or body mass index (BMI) for age *z*-scores of − 1 to − 1.9 corresponds to mild malnutrition, − 2 to − 2.9 to moderate malnutrition, and below − 3 indicates severe malnutrition [6]. In addition to food security, the poor nutritional status of a child can also be attributed to access to health services, poor feeding practices in a community, nutritional knowledge and awareness, and quantity and frequency of the consumed food [7, 8].

Positive associations between maternal nutrition knowledge and child nutritional outcomes are well documented for young children. As a proven factor, mothers’ nutritional knowledge significantly affects children’s nutritional status regardless of the family’s income level [9]. Additionally, parents’ knowledge and awareness about children’s nutrition is a more potent risk factor for child malnutrition than the availability of health centers [10]. The low health literacy of mothers causes them to have a poor understanding of children’s health problems and limits their interaction with the healthcare system [11]. Also, it seems that mothers with low health literacy do not clearly understand their children’s nutrition and malnutrition status and, therefore, cannot meet their children’s nutritional needs [12].

Meanwhile, one critical factor impacting people’s access to medical centers in developing countries is the distance of families to medical centers [13]. As a result, some patients may not be able to receive continuous health services due to the distance to the medical center or high transportation costs [14]. Also, it is imperative to note that increasing the distance and time to reach health centers is related to increasing malnutrition [15]. Health and treatment services can now be provided anywhere in the world through information and communication technology and are no longer limited by location. Technology has played an important role in improving health delivery systems, especially in disease control, diagnosis, and patient management. Technology can also be utilized to provide continuous education to patients [14]. In the past decade, smartphone ownership has surged, reaching at least half of the world’s population [16]; hence, smartphones could be used to enhance nutritional knowledge [17].

Smartphone-based technologies have the potential to significantly reduce costs along with providing better results [18]. Parents’ participation in child nutrition interventions can be more effective by using mobile apps to educate them about nutrition [19, 20]. The availability of mobile applications on nutrition education allows patients to follow healthy nutritional interventions even long after completing the treatment. Correspondingly, they reduce the rate of returning from the intervention program and the recurrence of health problems, thus helping to maintain healthy behavior in the long term [21].

A randomized controlled trial (RCT) protocol for *MyKid’sNutrition* is presented in this report, including information on the study’s intervention, design, procedures, and outcome measures. The intervention aims to elevate positive changes in maternal feeding practices and preschool children’s nutritional status by delivering educational content via an app.

### Objectives {7}

In this RCT, the primary objective is to investigate the effectiveness of *MyKid’sNutrition* app on children’s nutritional status and maternal nutritional knowledge, attitude, and practices.

### Trial design {8}

**Fig. 1:**
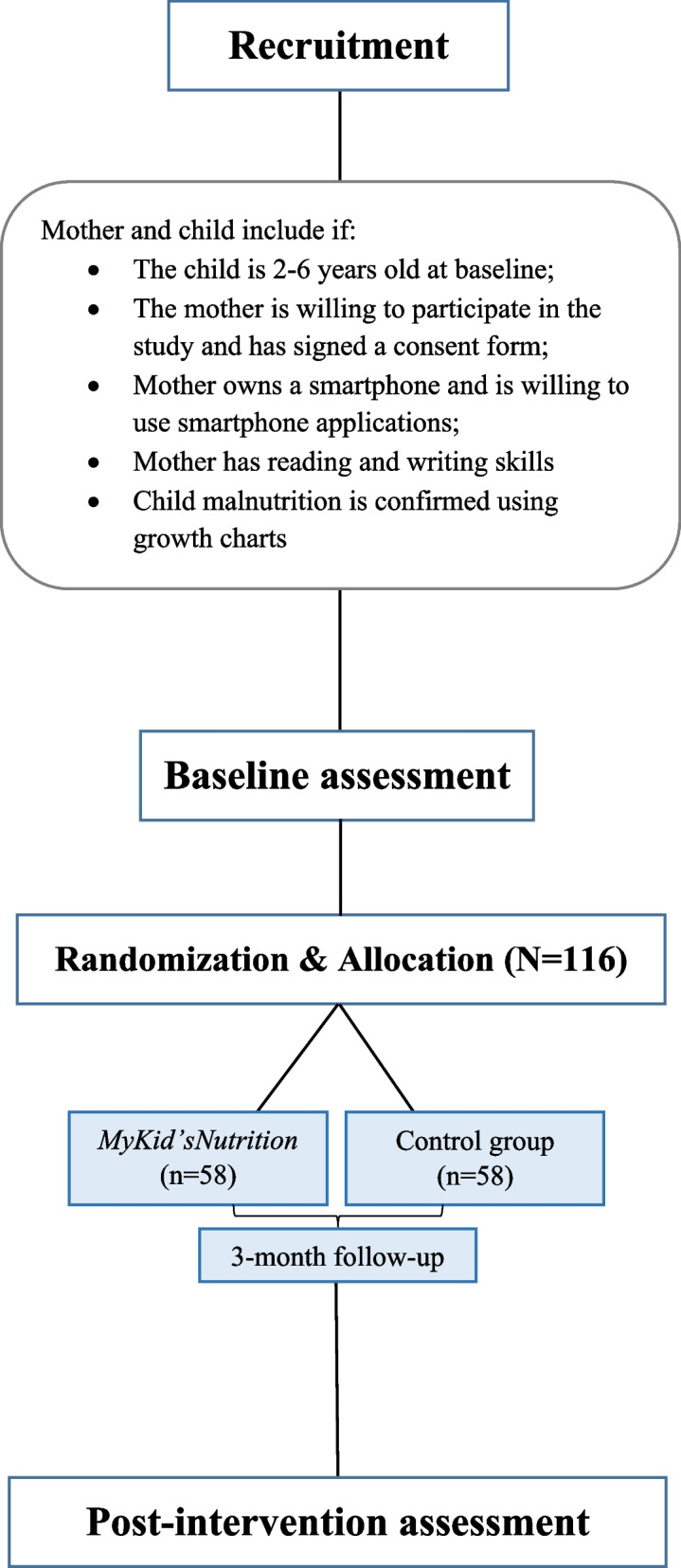
Flow chart of *MyKid’sNutrition* RCT study

We designed a two-arm RCT to evaluate the efficacy of the *MyKid’sNutrition* app. Following a baseline evaluation, children and their mothers will be randomly allocated to either the experimental group, *MyKid’sNutrition* app in addition to treatment as usual (TAU), or TAU alone as the control group. The patient allocation ratio will be 1:1. Figure 1 represents the *MyKid’sNutrition* RCT flowchart.

## Methods

### Participants, interventions, and outcomes

#### Study setting {9}

*MyKid’sNutrition* trial will be conducted in Isfahan, Iran. The subjects will be recruited from the pediatric outpatient clinics according to the study’s inclusion criteria.

#### Eligibility criteria {10}

The inclusion criteria will be as follows: children aged 2–6 years at baseline; mothers’ willingness to participate in the study and signing a written consent form; having a smartphone and willingness to use a smartphone application; mothers’ reading and writing skills; and confirmed malnutrition and underweight using growth charts.

There will be two exclusion criteria as follows: subjects suffering from a specific disease that can affect the results and those reluctant to continue participation in the study.

#### Who will take informed consent? {26a}

After the clinical team’s initial assessment, the eligible individuals will receive oral information along with an informational flyer about the study. After describing the research aims to participants and reminding that their participation is voluntary, the participants will sign written informed consent.

#### Additional consent provisions for collection and use of participant data and biological specimens {26b}

The consent form will inquire whether participants consent to the use of their data in the event of withdrawal from the trial or share relevant data with people from the universities taking part in the research or from regulatory authorities, where relevant. This trial will not involve collecting biological specimens for storage.

### Interventions

#### Explanation for the choice of comparators {6b}

This trial will evaluate the effect of *MyKid’sNutrition* mobile application on mothers’ nutritional knowledge and status of preschool children. The control group will receive standard care, which is treatment as usual.

#### Intervention description {11a}

After recruitment into the study, the subjects will be randomized into two separate arms. The participants in the intervention group will receive *MyKid’sNutrition* application, which will provide educational contents on children’s nutrition and weight status. The duration of the intervention will be three months in total. Both groups will receive standard care, which consists of treatment as usual for undernutrition.

### Development of the *MyKid’sNutrition* application

To deliver all the educational content, we designed and developed an application in Persian, the *MyKid’sNutrition* app, which can be installed on a mobile device or accessed via a web browser. The app includes contents of children’s interactive growth charts, textual and multimedia materials on healthy eating education, and contents specific for improving underweight children’s nutritional status.

**Fig. 2:**
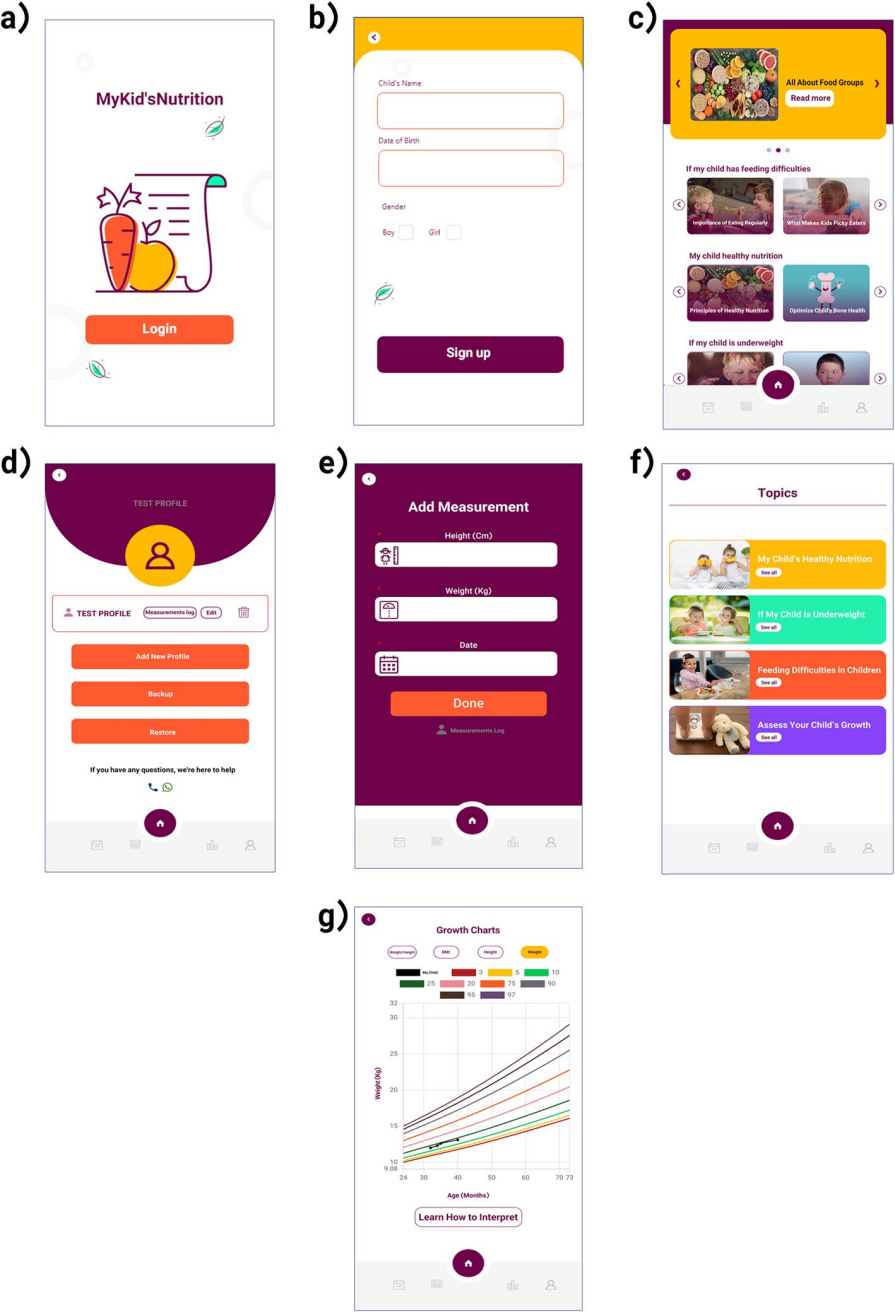
Translated interface of *MyKid’sNutrition* app: **a** login page; **b** sign-up page, which appears in the first use; **c** home page; **d** user’s profile page; **e** add measurements page; **f** topics page, each topic contains several contents; and **g** growth charts page, which demonstrates weight for age, height for age, BMI for age, and weight for height charts

All participants will have access to the *MyKid’sNutrition* app for free. While designing the application, we attempted to make it an attractive and user-friendly platform. *MyKid’sNutrition* app has five main screens. On the application’s home page, random contents from all available categories will be displayed with attractive and practical titles about improving children’s nutrition. On the user’s profile page, all mothers will be able to see their children’s information (such as name, date of birth, and gender) and edit it if needed. Also, in this section, the participants will be able to view and edit the application’s history of height and weight records. It will also have features like adding a new child profile, cloud data backup and restore, and contact technical support. On the add measurements page, the participants will be able to enter height and weight of the child to display them on the growth charts. In this section, the users will be able to input the weight recorded in the child’s past and see the child’s growth pattern. The topics page will list contents in four categories: (1) children’s healthy eating, (2) underweight children, (3) children’s loss of appetite, and (4) assessing child growth. Finally, different growth charts will be available on the growth charts page, and the child’s growth patterns will be shown based on the weights entered. Figure 2 depicts the app’s features and user interface.

The *MyKid’sNutrition* app was designed and developed by a team of nutritionists, pediatricians, designers, and developers. In the process that lasted for about one year, nutritionists and pediatricians initially provided the desired and required features to the design and programming team. A prototype of the application was designed, then existing problems were reported to the development team. After several upgrades, the final version of the application was eventually prepared. In order to prepare the content of the app, a team of nutritionists and pediatricians discussed nutrition educational content in numerous meetings derived entirely from scientific references. After the necessary corrections, the final version of the educational content was used in the application. User-manual of *MyKid’sNutrition* app is provided as Supplementary material 1.

### Content validity

To assess the quantification of content validity, we selected a Content Evaluation Panel consisting of ten content experts and ten lay experts. While the lay experts were potential research subjects, content experts were professionals with research experience or work in the field. Panel members were asked to judge the content validity ratio (CVR) and the content validity index (CVI). We listed the *MyKid’sNutrition* app’s content and provided it to the panel of experts as an evaluation list along with the printed content. The CVR was determined by asking each panelist, independently from the others, to indicate whether each item was “essential,” “useful,” or “not necessary.” The responses of all panelists were pooled to determine the percentage of items considered as “essential.” CVR was defined as (nE—n/2)/(n/2), where Ne was the number of panelists who indicated “essential” and N was the total number of panelists. Lawshe’s table was used to determine whether the CVR was approved [22]. The number of panelists in our study was 20 people, and content validity of all items was approved (CVR > 0.42).

To check the CVI, three criteria of simplicity, relevance, and clarity were analyzed separately on a 4-part Likert scale for each item by a panel of experts. CVI was computed by dividing the number of experts giving a rating of 3 or 4 by the total number of experts. Items were accepted based on a CVI score above 0.80. Table 1 demonstrates the CVI scoring method [22, 23]. In our study, the average CVI for all contents was 0.95, 0.96, and 0.90 for simplicity, relevance, and clarity, respectively.

**Table 1:** Scoring method for measuring content validity

Scores	**Simplicity**	**Relevance**	**Clarity**
1	Not simple	Not relevant	Not clear
2	Item need some revision	Item need some revision	Item need some revision
3	Simple but need minor revision	Relevant but need minor revision	Clear but need minor revision
4	Very simple	Very relevant	Very clear

#### Criteria for discontinuing or modifying allocated interventions {11b}

All subjects will be free to leave the study at any phase of the study for any reason without any consequences. Also, the research team will be free to remove any subject if they are uncooperative or lost to follow-up.

#### Strategies to improve adherence to interventions {11c}

The research team will contact subjects regularly to solve any probable problems in a timely manner and increase the subjects’ compliance.

#### Relevant concomitant care permitted or prohibited during the trial {11d}

There will be no restrictions on the relevant concomitant care. In addition, all participants will be free to continue with their usual care.

#### Provisions for post-trial care {30}

No specific post-trial care is planned, as the study is a low-risk intervention. At the end of the study, all participants will be offered a report of their children’s nutritional status from the post-assessment, which will determine whether the children need any specific treatment.

#### Outcomes {12}

The outcomes of *MyKid’sNutrition* trial would be the change in the growth indicators and mothers’ nutritional knowledge, attitude and practice within the groups, and the differences between the groups. Weight for height and BMI for age *z*-scores, which are the growth indicators and mothers’ nutritional literacy, will be measured at baseline and after 3-month follow-up visits. Additionally, the participants’ demographic and socio-economic status will be measured.

#### Participant timeline {13}

**Fig. 3:**
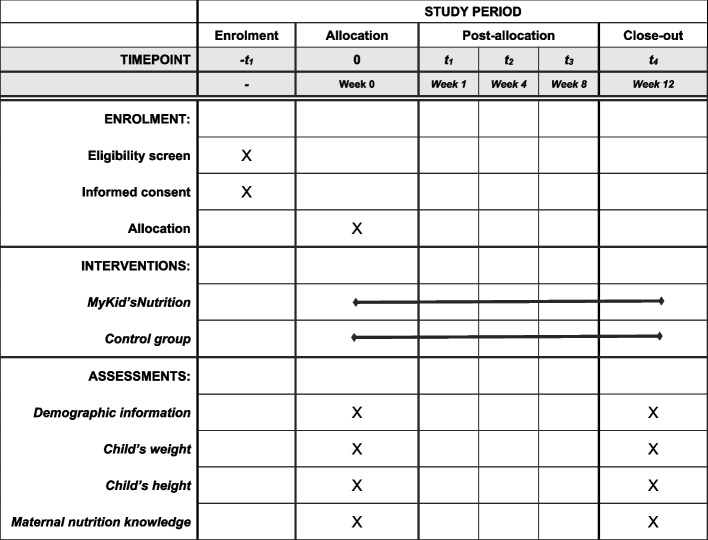
*MyKid’sNutrition* RCT study schedule of enrolment, interventions, and assessments (according to SPIRIT guidelines; Supplementary Material 2)

Figure 3 shows the *MyKid’sNutrition* RCT study schedule of enrolment, interventions, and assessments (according to SPIRIT guidelines; Supplementary Material 2).

#### Sample size {14}

To estimate the sample size, we performed a statistical power analysis based on previous studies [24] and considering an alpha equal to 0.05 and a power of 0.80 with an effect size of 0.5 using G*Power 3.1.9.7. Accordingly, the sample size was estimated as 102 participants (51 mother–child pairs in each group). Finally, considering a dropout rate of 14%, the total sample size was calculated as 116 people.

#### Recruitment {15}

All subjects will be selected from people referring to the pediatric outpatient clinics in Isfahan, Iran. We will reach out to participants who attend in-person visits. We will also use written advertising (flyers, brochures, etc.). All potential and interested volunteers will be referred to a coordinator who will determine if the individual meets the initial eligibility requirements. Finally, all eligible participants will sign a written informed consent and agree to participate in the study voluntarily.

### Assignment of interventions: allocation

#### Sequence generation {16a}

Following baseline assessment, all eligible participants will be randomized (1:1 ratio) to intervention or usual care groups. The process of random sequence generation and the allocation of participants to intervention conditions will be implemented on an online platform by an independent researcher not involved in the process of subject recruitment.

#### Concealment mechanism {16b}

Since the randomization process occurs using an online tool and by an independent researcher, the process is fully concealed from both study investigators and prospective participants until the study arm is assigned.

#### Implementation {16c}

A clinical research coordinator will enroll all participants and assign them into groups.

### Assignment of interventions: blinding

#### Who will be blinded {17a}

Due to the nature of the intervention, neither the research team nor the patients in both arms can be blinded to the patients’ allocation. However, the outcome assessment team, as well as those who are involved in data analysis and statistics will be blinded to the group allocations.

#### Procedure for unblinding if needed {17b}

The design is open-label with only outcome assessors being blinded so unblinding will not occur.

### Data collection and management

#### Plans for assessment and collection of outcomes {18a}

We will use a questionnaire to collect all participants’ sociodemographic and baseline information, including name, sex, age, contact information, family size and income, past medical history, phone number(s), education, and occupation. The research team will measure all children’s anthropometric indices and the presence of undernutrition will be assessed based on growth charts [6]. Using a questionnaire with confirmed psychometric properties, the mothers’ nutritional knowledge, attitude, and practice will be assessed [25]. All data will be collected in a uniform and reproducible manner by trained data collectors. The quality of the data will be supervised by the principal investigator.

#### Plans to promote participant retention and complete follow-up {18b}

The research team will provide periodic communications with participants via telephone and text message. Also, they will use methods to improve participant retention, such as reminders for using the application and scheduling appointments for post-intervention visit.

#### Data management {19}

To improve the quality and accuracy of data, all data will be entered twice and double-checked by two independent administrators. The data obtained in this study will be archived at Tabriz University of Medical Sciences, Iran. Access to this data will be limited to data administrators and statisticians. Each patient will be assigned a unique and confidential participant ID that is only accessible by the research team, and all personal data will be coded. We will separate patients’ personal information from the database containing their medical information.

#### Confidentiality {27}

Using a study identification code for each participant, the research data will be stored. The research team will have access to the key to the identification code list during the study, which will be documented and safeguarded by the principal investigator according to the research guidelines after the completion of the study. Personal details of the participants will not be reported in the publications.

#### Plans for collection, laboratory evaluation, and storage of biological specimens for genetic or molecular analysis in this trial/future use {33}

This is not applicable to the current study, since this trial does not involve collecting biological specimens for storage.

### Statistical methods

#### Statistical methods for primary and secondary outcomes {20a}

We will use descriptive statistics for summarizing and determining the sample’s characteristics and distribution among the patients. Frequencies and proportions will be used to summarize categorical data. Analyzing associations between qualitative data variables will be done using the chi-square (*χ*^2^) test or Fisher’s exact test, as appropriate. The unpaired *t*-test or Mann–Whitney *U* test will be used to analyze quantitative data between the two independent groups. The analyses will be adjusted for age, sex, and other baseline characteristics as covariates when needed. A 95% confidence interval will be provided for all comparative analyses, and statistical significance for main effects will be determined as 5%.

#### Interim analyses {21b}

There are no interim analyses planned.

#### Methods for additional analyses (e.g., subgroup analyses) {20b}

There are no subgroup analyses planned.

#### Methods in analysis to handle protocol non-adherence and any statistical methods to handle missing data {20c}

Missing data will not be imputed. All available data will be used in the models.

#### Plans to give access to the full protocol, participant-level data, and statistical code {31c}

The datasets used and/or analyzed during the current study can be made available by the corresponding author upon reasonable request and in agreement with the research collaboration and data transfer guidelines of the Tabriz University of Medical Sciences, as is the full protocol.

### Oversight and monitoring

#### Composition of the coordinating center and trial steering committee {5d}

The principal investigator is the designer of the trial and responsible for conducting the research. The principal investigator and research physicians are responsible for the recruitment, treatment, and follow-up of the participants. The technology development research fellow is responsible for the design and development of the study application. The staff responsible for recruitment activities are responsible for selecting eligible subjects, ensuring that the recruitment process adheres to the defined criteria and protocols. To supervise the entire study process, a steering committee will be set up. Also, the study team will be in continuous communication to monitor the study’s conduct.

#### Composition of the data monitoring committee, its role, and reporting structure {21a}

Since the trial has a short duration and the treatment regimens are associated with known minimal risks, no data monitoring committee will be established.

#### Adverse event reporting and harms {22}

There will be no adverse events or harm in this study due to the nature of the intervention.

#### Frequency and plans for auditing trial conduct {23}

We will send a report to the auditor every 6 months.

#### Plans for communicating important protocol amendments to relevant parties (e.g., trial participants, ethical committees) {25}

All protocol changes will be approved by the Ethics Committees of Tabriz University of Medical Sciences, Iran. In case amendments concern or affect participants in any way, they will be informed about the changes. If needed, additional consent will be requested and registered. Also, online trial registries will be updated accordingly.

#### Dissemination plans {31a}

All findings from the RCT study will be presented at national and international scientific meetings in nutrition, pediatrics, parenting, and other related conferences. A detailed report of the research will also appear in peer-reviewed journals. Additionally, the study results will be shared with participants, healthcare professionals, and healthcare providers.

### Patient public involvement

During the application designing phase and content validity assessment, we actively sought the cooperation and input of potential subjects, ensuring patient public involvement in the research. We engaged with individuals who could provide valuable perspectives and insights, allowing us to develop an application that is user-friendly and relevant to their needs. Their involvement played a crucial role in shaping the design and content of the application, ensuring its validity and appropriateness.

## Discussion

Mobility is crucial to the concept of participatory healthcare. It liberates healthcare professionals and receivers from temporal and spatial restrictions, facilitating their healthcare engagement. Not unexpectedly, the field of mobile health, or mHealth as it is widely called, has significantly received interest over the past few years as its use has spread throughout the healthcare business. The mobility concept has evolved from the physical transportation of healthcare personnel and equipment to straightforward information transport using modern technologies. This novel paradigm begins with telemedicine and telehealth and gives rise to the concept of eHealth, of which mHealth is a subset. Smartphones and related technology represent the next step in the evolution of “transporting information to alter healthcare” and, subsequently, mobility and involvement in healthcare [26].

Considering their high availability in the society, using smartphones can help to deliver educational content on a large scale and at a low cost. Mobile phone applications provide new opportunities for patients to receive support. Research on these types of interventions, particularly within the pediatric nutrition field, is extremely valuable. Little is known about conducting clinical trials of mHealth interventions in Iran, specifically in the pediatric field, and the progress of this study will contribute to the further evolution of research in this field in the future.

*MyKid’sNutrition* has been designed to be a comprehensive, inexpensive, and user-friendly resource for parents, particularly mothers, who wish to improve their feeding practices and instill a healthy diet in young children. Along with dietary solutions for underweight, loss of appetite, and malnutrition in children, the application can teach the mother how to behave with the child in a practical and detailed manner.

To the best of our knowledge, this is one of the first initiatives to design and evaluate an application for improving and controlling childhood malnutrition in Iran. Even among lower socioeconomic groups, smartphone usage continues to grow, and a mobile tool that can educate patients and increase treatment adherence may be a cost-effective solution to the increased costs resulting from face-to-face meetings with health professionals.

## Trial status

The study began on October 23, 2021, and is expected to be completed by July 10, 2023. The recruitment process began on October 31, 2022, and is expected to end by March 31, 2023.

### Supplementary Information

**Additional file 1:** **Supplementary material 1.** Spirit Checklist; Supplementary material 2. User-manual of *MyKid’sNutrition* app.

## Data Availability

The datasets used and/or analyzed during the current study are available from the corresponding author on reasonable request.

## Abbreviations

WHO: World Health Organization
BMI: Body mass index
RCT: Randomized controlled trial
TAU: Treatment as usual
CVR: Content validity ratio
CVI: Content validity index
*mHealth*: Mobile health
