# Supplementary material for: MyKid’sNutrition mobile application trial: a randomized controlled trial to promote mothers’ nutritional knowledge and nutritional status of preschool children with undernutrition—a study protocol
Source: Trials. 2023 Aug 19;24:544. doi: 10.1186/s13063-023-07503-w (PMC10439575; doi:10.1186/s13063-023-07503-w)
Supplement: Supplementary file 1 — Additional file 1: Supplementary material 1. Spirit Checklist; Supplementary material 2. User-manual of MyKid’sNutrition app. [file 13063_2023_7503_MOESM1_ESM.zip › Supplementary Material 2.pdf]

MyKid’sNutrition

A mobile application to promote mothers' nutritional knowledge and nutritional status of preschool children with undernutrition

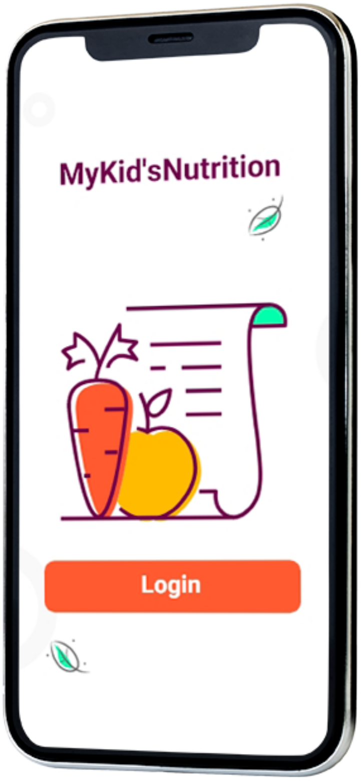

User Manual

This version is an English translation of the user manual for the application provided to participants in Persian.

Sign Up

When you first launch the application, you will be prompted to enter your child's information, such as their name, date of birth, and gender. Please provide these details to proceed with using the app.

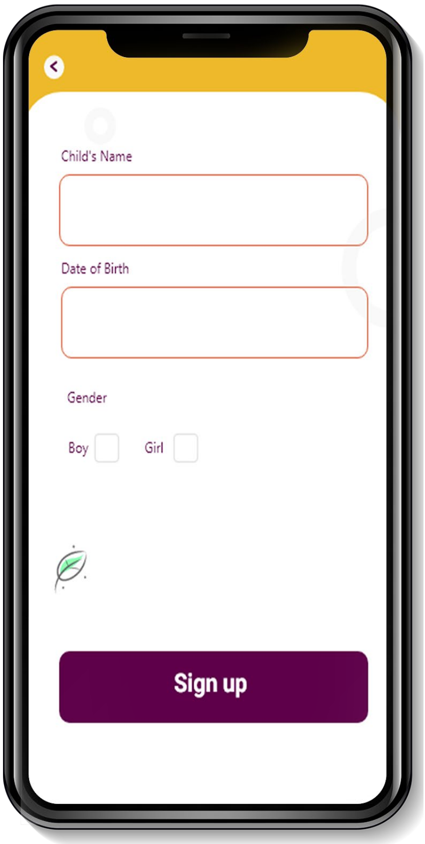

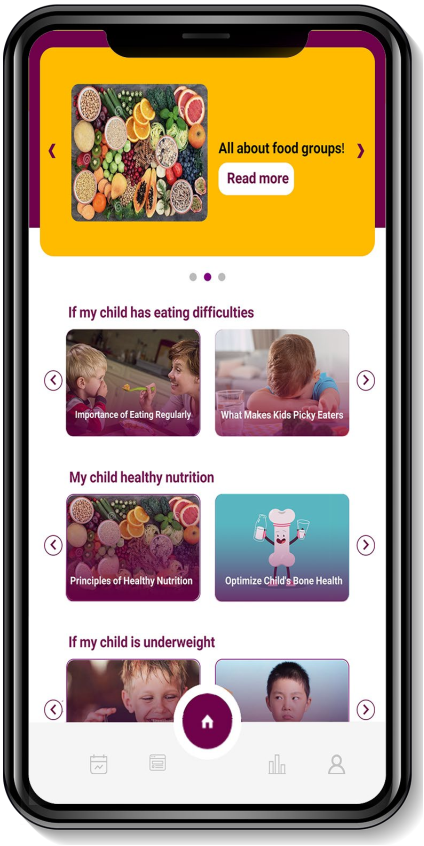

Home Page

On the home page, you will find a selection of suggested educational content organized into different categories. By selecting any of them, you will be able to view the full text.

Topics

In this section, marked with a red circle, you can access educational content organized into four categories. By selecting each category, a list of all available content will be presented to you. To read the full articles, simply click on the 'Read More' option. It is recommended to gradually go through all the content available in this section.

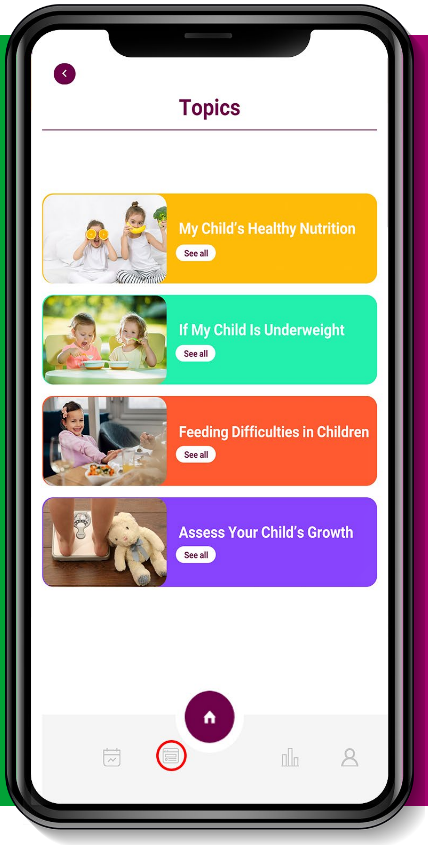

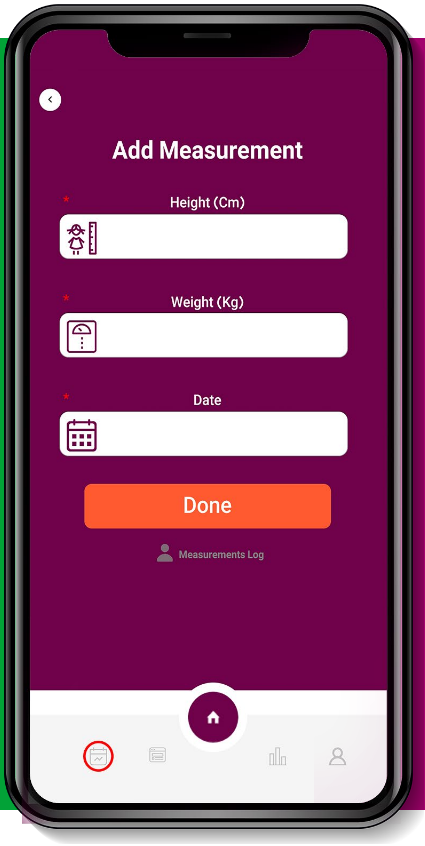

Add Measurements

In this section, marked with a red circle, enter your child's height and weight after each measurement. You can also enter previous measurements along with their respective dates. The application will use this information to generate and display a growth chart for your child. A sample of this chart will be available for you to view on the next page.

Growth Charts

To access the growth charts, please select the highlighted key button indicated by the red circle. These charts are based on your child's age, height, and weight. Within this section, you will find various types of growth charts, and detailed tutorials are available to help you understand each of them. To view the tutorial related to a specific chart and learn how to interpret it, simply use the 'Learn How to Interpret' option provided below the chart.

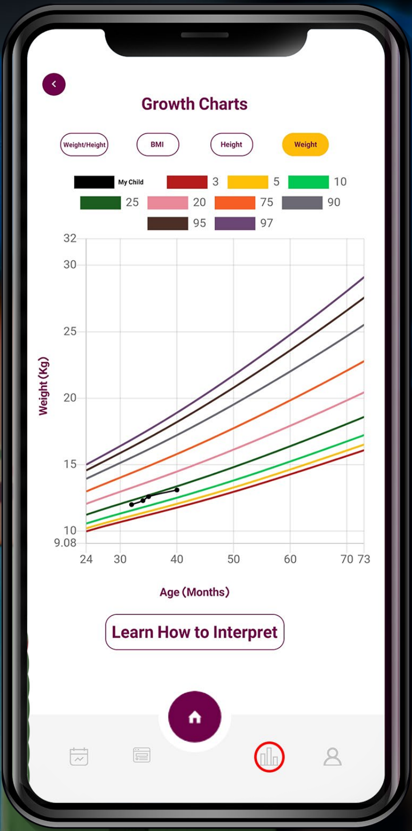

Child's Profile

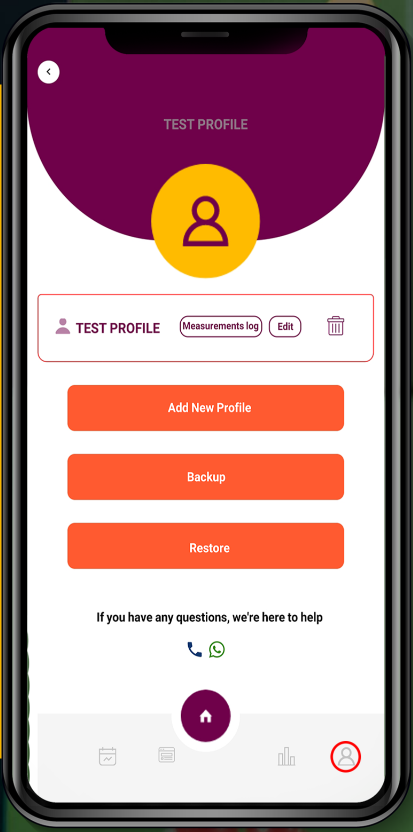

On the child's profile page, indicated by the red circle, you can edit your child's information, such as their name, date of birth, and gender. You can also view and modify the history of height and weight measurements entered. Furthermore, this section provides the option to back up your child's information, ensuring that you can restore it when needed. Additionally, you can contact us through phone calls or text messages using this section.

MyKid’sNutrition

Please don't hesitate to contact us if you have any questions or need assistance;

WE ARE HERE TO HELP

+98 922 326 7088

hojati.a@tbzmed.ac.ir
